# Cognitive Impairments in Schizophrenia: A Study in a Large Clinical Sample Using Natural Language Processing

**DOI:** 10.3389/fdgth.2021.711941

**Published:** 2021-07-15

**Authors:** Aurelie Mascio, Robert Stewart, Riley Botelle, Marcus Williams, Luwaiza Mirza, Rashmi Patel, Thomas Pollak, Richard Dobson, Angus Roberts

**Affiliations:** ^1^Department of Biostatistics and Health Informatics, Institute of Psychiatry, Psychology and Neuroscience, King's College London, London, United Kingdom; ^2^National Institute for Health Research (NIHR) Biomedical Research Centre at South London and Maudsley National Health Service (NHS) Foundation Trust and King's College London, London, United Kingdom; ^3^Health Data Research UK, London, United Kingdom; ^4^GKT School of Medical Education, King's College London, London, United Kingdom; ^5^Department of Psychosis Studies, Institute of Psychiatry, Psychology and Neuroscience, King's College London, London, United Kingdom

**Keywords:** natural language processing, electronic health records, cognition, schizophrenia, data mining

## Abstract

**Background:** Cognitive impairments are a neglected aspect of schizophrenia despite being a major factor of poor functional outcome. They are usually measured using various rating scales, however, these necessitate trained practitioners and are rarely routinely applied in clinical settings. Recent advances in natural language processing techniques allow us to extract such information from unstructured portions of text at a large scale and in a cost effective manner. We aimed to identify cognitive problems in the clinical records of a large sample of patients with schizophrenia, and assess their association with clinical outcomes.

**Methods:** We developed a natural language processing based application identifying cognitive dysfunctions from the free text of medical records, and assessed its performance against a rating scale widely used in the United Kingdom, the cognitive component of the Health of the Nation Outcome Scales (HoNOS). Furthermore, we analyzed cognitive trajectories over the course of patient treatment, and evaluated their relationship with various socio-demographic factors and clinical outcomes.

**Results:** We found a high prevalence of cognitive impairments in patients with schizophrenia, and a strong correlation with several socio-demographic factors (gender, education, ethnicity, marital status, and employment) as well as adverse clinical outcomes. Results obtained from the free text were broadly in line with those obtained using the HoNOS subscale, and shed light on additional associations, notably related to attention and social impairments for patients with higher education.

**Conclusions:** Our findings demonstrate that cognitive problems are common in patients with schizophrenia, can be reliably extracted from clinical records using natural language processing, and are associated with adverse clinical outcomes. Harvesting the free text from medical records provides a larger coverage in contrast to neurocognitive batteries or rating scales, and access to additional socio-demographic and clinical variables. Text mining tools can therefore facilitate large scale patient screening and early symptoms detection, and ultimately help inform clinical decisions.

## Introduction

Despite the relatively limited prevalence of schizophrenia (Sz) worldwide (around 1% of the population), it is one of the largest contributors to the global burden of disease, ranking in the top 10 illnesses (1). Schizophrenia is associated with poor lifestyle (e.g., obesity, smoking) and high suicide rates (2), leading to an estimated reduction in life expectancy by 10–25 years (3). Additionally, Sz is a major cause of disability, ranked third after blindness and paraplegia, resulting in a significant economic and human cost (4).

In particular, cognitive dysfunctions have been associated with poor functional outcomes, and are present in most patients, impairing their ability to establish social interactions, plan, adapt, or solve problems (5). Cognitive dysfunctions in Sz have been extensively studied and documented (6, 7) and typically include impairments in memory, attention, executive functioning, learning, and to a lesser extent social cognition (8).

Over the last decade, several neurocognitive assessment batteries such as the MATRICS Consensus Cognitive Battery (MCCB) (9) or the Cambridge Neuropsychological Test Automated Battery (CANTAB) (10) have been increasingly utilized in the United Kingdom (UK) as outcome measures for clinical trials targeting cognition in schizophrenia (Sz) (11).

Whilst such neuropsychological tests are valuable tools, they remain relatively expensive, may require several hours to administer, and are not routinely applied in clinical practice (12, 13).

Conversely, natural language processing (NLP) allows the harvesting of rich clinical data recorded in the free-text narratives of Electronic Health Records (EHRs), therefore offering an alternative method to derive detailed clinical phenotyping on large volumes of clinical data. NLP has thus far been used to process and analyse information from the unstructured text of EHRs for a variety of healthcare applications, including patients identification for trials, de-identification of records, extraction of disease risk factors or symptoms, or care and cost evaluation (14). Looking more specifically at symptom classification, a recent systematic literature review identified 14 studies presenting symptom information extraction as a primary measure (15), including adverse drug events, depressed mood and mood instability for mental healthcare (16, 17). Several additional studies targeting Sz were also identified: (18–20) detected, respectively, mentions of negative symptoms, poor insight and atypical hallucinations (21), identified abnormal motor behavior, delusion, disorganized thinking, hallucination and negative symptoms. However, to the best of our knowledge, no previous work has focused on extracting cognitive impairments from the unstructured portion of medical records.

In this study, we present a novel NLP tool that identifies cognitive impairments for patients with Sz from the unstructured portions of EHRs. We demonstrate the benefit of using an NLP approach by evaluating it against a routinely administered rating scale, and using the information extracted from free text to explore the association between cognitive problems, socio-demographic factors and clinical outcomes.

## Materials and Methods

All the data used in this study was extracted from the South London (UK) and Maudsley NHS Foundation Trust Biomedical Research Center Case Register (CRIS) comprising pseudonymized EHRs of over 350,000 patients (22, 23).

### Cognitive Impairments Definition

In order to define the scope of cognitive impairments, we reviewed the domains covered by selected major cognitive test batteries and scales that were specifically developed for or adapted to Sz, including MCCB (9), CANTAB (10), The Brief Assessment of Cognition in Schizophrenia (BACS) (13), the Schizophrenia Cognition Rating Scale (SCoRS) (24), and the Subjective Scale to Investigate Cognition in Schizophrenia (SSTICS) (25).

Several consistent major domains of impairments emerged from this analysis, namely: attention, memory, executive functioning, and social cognition, which were then used to implement the NLP framework.

### Measurement Development

We used NLP to identify mentions of cognitive impairments in the unstructured portion of clinical records.

#### Annotations Dataset

A set of keywords relevant to cognition, spanning the different domains identified (attention, memory, executive functioning, social cognition, and general cognitive impairments) was derived manually from SNOMED-CT (26) and the Unified Medical Language System (27) terminologies, and further enriched with clinicians' feedback.

Fourteen thousand sentences containing these keywords were randomly sampled from documents of patients diagnosed with Sz and active in CRIS between 2007 and 2020. The sentences were then annotated by two medical students for the presence of symptoms (indicating that the impairment is affirmed and relates to the patient, e.g., “ZZZ shows poor concentration,” as opposed to negated or irrelevant mention, e.g., “ZZZ shows good concentration” or “dose adjusted to a serum-lithium concentration of 0.4–1 mmol/L”). The annotations were conducted in a single phase using detailed guidelines designed in conjunction with researchers and clinicians, achieving an overall inter-annotator agreement of 93.7%.

### Classification Model

Following the comparative analysis of algorithms for biomedical text classification tasks conducted in Mascio et al. (28), we fine-tuned a transformers-based model, BioBERT (29, 30) using the annotated dataset (code available on *github*).

**Figure 1:**
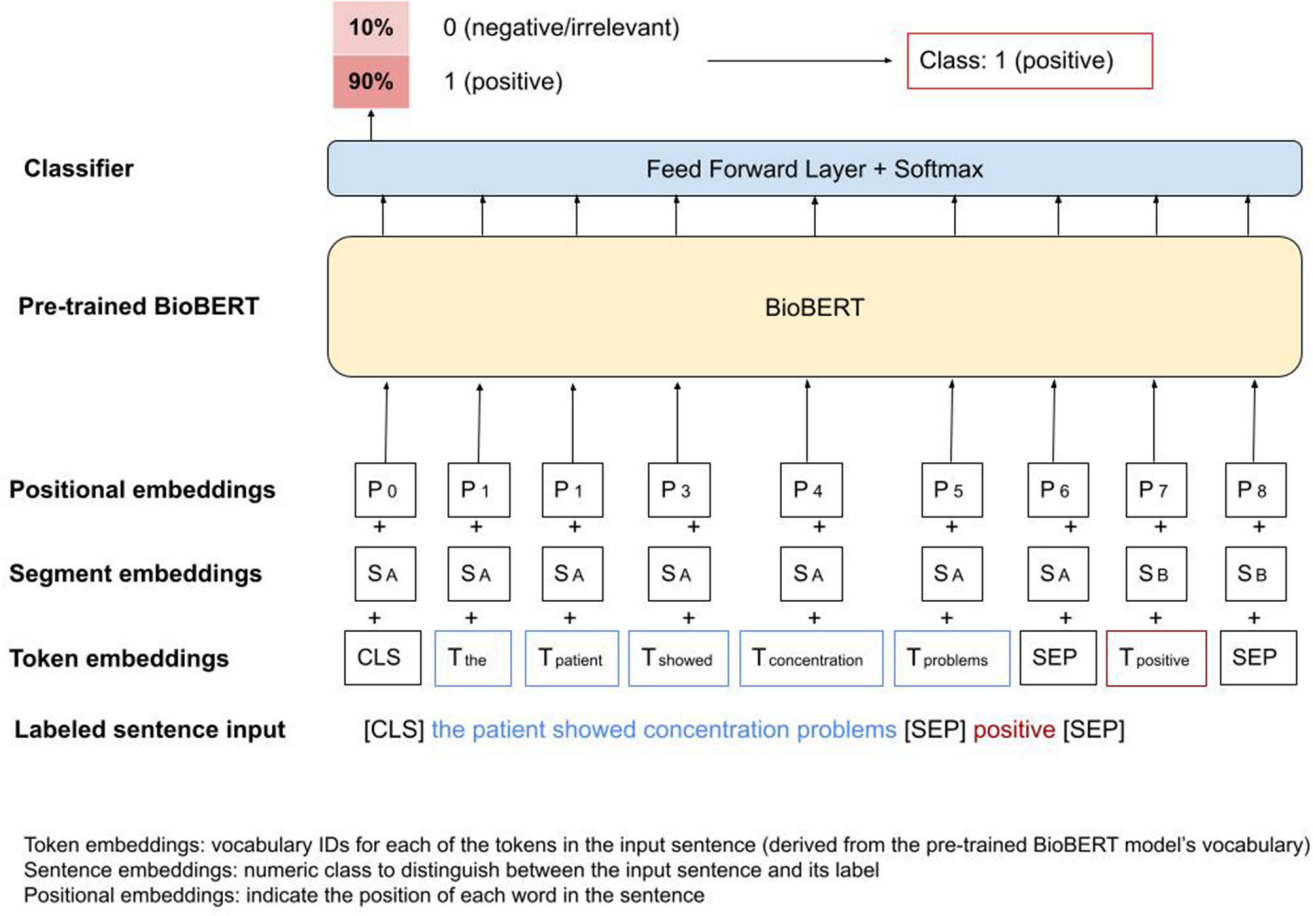
BioBERT fine-tuning.

BioBERT is a transformers-based model pre-trained on large scale biomedical corpora (31), and consequently already captures medical terminologies and jargon. In order to fine-tune BioBERT to the cognitive symptoms classification task, we used the pre-trained model, added an untrained softmax classification layer at the end, and trained the new model for our task (as illustrated in Figure 1 below) (32).

We used F1 score, precision and recall to estimate the model's performance (33). Precision here measures the number of positive predictions that actually correspond to positive examples [True Positives/(True Positives + False Positives)], and recall measures the number of positive examples correctly predicted as positive [True Positives/(True Positives + False Negatives)]. F1 score balances both precision and recall in one single metric: F1 = 2 × precision × recall/(precision + recall).

#### Cognitive Scores

The symptoms extracted via the fine-tuned NLP model were used to derive a composite score of cognitive impairment (“NLP-CI”), in the following way:

1. For a given patient at a given point in time, a score of 1 is assigned if a cognitive problem is recorded in any document with that date. This process is repeated for each of the five individual cognitive domains (attention, memory, executive functioning, social cognition, and general cognitive problem), generating a set of five binary scores.
2. The NLP-CI is computed by summing the scores across all individual domains, resulting in a rating scale between 0 and 5, and denoting the number of cognitive symptoms exhibited by the patients at a given date. We also defined its associated binary variable on the basis of a score of 1 or more (“NLP-CI-b”).

A patient will then be assigned, for each date at which documents were recorded, five binary scores indicating the presence or absence of individual cognitive problems, as well as two composite scores indicating the number of different symptoms recorded and the presence of more than one symptom.

### Comparison With HoNOS

The Health of the Nation Outcome Scales (HoNOS) has been developed to assess the health and functioning of working age adults with severe mental illness (34), and is routinely administered in most mental health services in the UK (35). HoNOS comprises 12 scales and, of specific interest to our study, a cognitive impairments subscale (“HoNOS-CI”) which aims at measuring cognitive problems severity. This subscale ranges from 0 to 4, 0 meaning no problem, 1,2,3,4 indicating minor, mild, moderate, and severe problems (34).

Despite the fact that the NLP-CI score evaluates the numbers of cognitive domains impaired (as opposed to measuring general cognitive impairment severity) and coverage differences (HoNOS is not administered to children, and does not encompass all cognitive domains), the systematic and frequent use of that rating scale in CRIS makes it an ideal proxy to compare with the NLP-CI score. For this subscale we derived a binary variable of a score of 1 or more (“HoNOS-CI-b”), in order to replicate best NLP-CI-b that potentially includes non-clinically significant impairments.

### Participants and Clinical Data

Two samples were generated from CRIS for analysis:

1. **Sample A (*n* = 24,614, 214,239 observations):** patients with Sz active in CRIS between 2007 and 2020. This sample was used to analyze the prevalence of cognitive symptoms extracted by the NLP tool, along various demographic and clinical measures including age, gender, marital status, ethnicity, education, first language, employment, comorbidity with dementia, use of antipsychotic, antidepressant, and antidementia medications [following the method used in Bendayan et al. (36)]. We also used the following HoNOS subscales as covariates: “problems with activities of daily living” (HoNOS-ADL), “problems with relationships” (HoNOS-social), “problems associated with hallucinations and delusions” (HoNOS-positive symptoms), and “problems with depressed mood” (HoNOS-depression) (18). For all these HoNOS subscales, we derived binary variables based on a score of 2 or more, indicating clinically significant impairment (34).
2. **Sample B (*n* = 12,234, 116,719 observations):** the subset of patients from sample A who had at least 3 HoNOS scores recorded during their total length of care, following (37). This sample was used to model HoNOS trajectories and compare associations of cognition with demographic factors and clinical outcomes for both HoNOS and the NLP metric.

For each sample, the data was aggregated using 6 months buckets in order to derive the analyses (36).

### Statistical Analysis

For both NLP-CI and HoNOS-CI scales, a threshold score of 0 was applied to measure the presence or absence of cognitive impairments (indicating that at least some level of cognitive dysfunction was recorded). This binary scale was used in conjunction with the ordinal scale to conduct analyses.

Logistic and linear regressions were used to assess the association of sociodemographic factors and clinical outcomes with the presence or absence of cognitive impairments. For clinical outcomes, the relation between cognition and number of hospital admissions and length of admission within 6 months of the score was estimated using linear regression. For socio-demographic covariates, missing data was treated as a separate category, and those showing a too high proportion of missing data or too low associations with both NLP-CI and HoNOS-CI were dropped from the analyses.

Finally, grouping was performed for certain variables when found to increase association or to reduce imbalance of sub-categories. Sensitivity analyses were performed to compare grouping methodologies and the grouping maximizing the association was selected.

For trajectories modeling, mixed linear models were used, with random slope and intercept. Linear and quadratic unconditional models were also examined, with and without random coefficients, but dropped in preference for the model maximizing the log-likelihood.

## Results

### Performance of NLP-CI

The performance of the NLP tool was evaluated using 10-fold cross-validation, using the manually annotated sentences described in section Measurement development. Further testing was conducted using a newly generated sample of 800 sentences, which were again manually annotated and compared against BioBert's classification labels independently.

The results from the cross validation analysis (averages on all folds for test and training sets, using weighted metrics) are summarized in Table 1A.

**Table 1A:** Performance of the NLP-CI tool for individual domains (average over 10-fold cross validation).

		**Test set**			**Training set**		
		**(1,400 sentences)**			**(12,600 sentences)**		
**Symptom**	**Support**	**F1**	**P**	**R**	**F1**	**P**	**R**
Attention	2,800	93%	94%	83%	96%	97%	96%
Memory	2,800	96%	97%	96%	97%	98%	98%
Executive Function	2,800	93%	93%	93%	96%	96%	96%
Emotion	2,800	93%	93%	93%	93%	93%	93%
Other cognition	2,800	91%	91%	91%	98%	98%	98%
**Total**	14,000	93%	94%	93%	97%	97%	97%

*F1, F1-score; P, precision; R, recall*.

F1 scores ranged between 91 and 96% depending on the cognitive domains, and the overall application encompassing all symptoms showed a F1 score of 93%.

The independent blind testing, presented in Table 1B, showed similar performance results.

**Table 1B:** Performance of the NLP-CI tool for individual domains (separate blind set).

**Symptom**	**Support**	**F1**	**P**	**R**
Attention	160	97%	98%	96%
Memory	160	91%	86%	96%
Executive function	160	94%	88%	100%
Emotion	160	97%	100%	94%
Other cognition	160	92%	86%	100%
**Total**	**800**	**94%**	**92%**	**97%**

*F1, F1-score; P, precision; R, recall*.

### Prevalence of Cognitive Impairments and Cognitive Profiles

Table 2 shows the distribution of cognitive profiles by symptoms, as identified by NLP-CI. Of the 24,614 patients diagnosed with Sz in CRIS between 2007 and 2020, 55% had one or more mentions of cognitive problems in their clinical records, and 60% of patients with at least one problem recorded have mentions in all of the five domains identified. This supports the hypothesis that cognitive dysfunctions are a common feature of Sz.

**Table 2:** Cognitive impairments profiles (Sample A).

**Max NLP-CI recorded**	**Prevalence % (nb patients)**	**Most prevalent profile**
0	45% (11,069)	–
1	3% (825)	Executive function
2	4% (1,006)	Memory—executive function
3	6% (1,435)	Attention—memory—executive function
4	9% (2,270)	Attention—memory—executive function—other
5	33% (8,009)	Attention—memory—emotion—executive function—other
**Total**	100% (24,614)	–

The symptoms most frequently mentioned were executive function (present in 53% of patients from sample A), followed by memory (47%) and attention (44%).

### Association With Socio-Demographic Factors

We ran binary logistic regression models to analyze the association of cognitive impairments with various socio-demographic and clinical factors (Table 3).

**Table 3:** Analysis of factors associated with cognitive impairments in Sz (Sample B).

**Factor**	**Group**	**Sample B population 12,234 patients**				**Association with cognitive impairment**	
		**% (nb patients)**	**#scores**	**NLP-CI mean (SD)**	**HoNOS-CI mean (SD)**	**NLP-CI-b OR (95% CI)**	**HoNOS-CI-b OR (95% CI)**
**Age**	20–30	22% (2,654)	17,950	1.9 (1.9)	0.5 (0.8)	2.08 (0.03)^***^, [1.96, 2.2]	2.94 (0.03)^***^, [2.75, 3.13]
	30–40	22% (2,710)	25,177	1.7 (1.8)	0.5 (0.8)	2.08 (0.03)^***^, [1.97, 2.2]	2.8 (0.03)^***^, [2.63, 2.97]
	40–50	23% (2,815)	29,619	1.5 (1.7)	0.5 (0.8)	1.98 (0.03)^***^, [1.88, 2.09]	3.03 (0.03)^***^, [2.86, 3.22]
	50–60	13% (1,592)	22,422	1.4 (1.6)	0.7 (0.9)	1.89 (0.03)^***^, [1.79, 2]	3.75 (0.03)^***^, [3.53, 3.98]
	60–70	7% (894)	10,739	1.4 (1.6)	0.8 (1)	1.82 (0.03)^***^, [1.71, 1.93]	4.76 (0.03)^***^, [4.47, 5.08]
	70+	7% (852)	9,175	1.4 (1.6)	1.1 (1.1)	1.86 (0.03)^***^, [1.75, 1.97]	6.07 (0.03)^***^, [5.68, 6.47]
	15–20	6% (717)	1,637	1.8 (2.1)	0.6 (0.8)	**Reference**	**Reference**
**Gender**	Female	42% (5,160)	48,092	1.5 (1.7)	0.6 (0.9)	0.82 (0.01)^***^, [0.8, 0.84]	0.89 (0.01)^***^, [0.87, 0.91]
	Male	58% (7,074)	68,627	1.6 (1.7)	0.6 (0.9)	**Reference**	**Reference**
**Diagnosis**	Sz only	91% (11,078)	104,724	1.5 (1.7)	0.6 (0.8)	0.88 (0.02)^***^, [0.85, 0.91]	0.68 (0.02)^***^, [0.65, 0.71]
	Sz + dementia	9% (1,156)	11,995	1.8 (1.8)	1.1 (1.1)	**Reference**	**Reference**
**Education**	GCSE+	49% (6,000)	62,361	1.7 (1.8)	0.5 (0.8)	1.3 (0.01)^***^, [1.27, 1.33]	0.91 (0.01)^***^, [0.88, 0.93]
	no	51% (6,234)	54,358	1.5 (1.7)	0.7 (0.9)	**Reference**	**Reference**
**Ethnicity**	White	62% (7,556)	71,163	1.5 (1.7)	0.6 (0.9)	0.84 (0.01)^***^, [0.82, 0.86]	1.03 (0.01)*, [1, 1.05]
	Other	38% (4,678)	45,556	1.6 (1.7)	0.6 (0.8)	**Reference**	**Reference**
**Marital**	Married/cohabiting	11% (1,300)	11,114	1.2 (1.6)	0.6 (0.9)	0.72 (0.02)^***^, [0.69, 0.75]	0.85 (0.02)^***^, [0.82, 0.89]
**status**	Single/separated	89% (10,934)	105,605	1.4 (1.8)	0.6 (0.8)	**Reference**	**Reference**
**Employment**	Employed	5% (559)	5,003	1.4 (1.7)	0.4 (0.7)	0.8 (0.03)^***^, [0.76, 0.85]	0.81 (0.03)^***^, [0.76, 0.87]
	Other	95% (11,675)	111,716	1.5 (1.7)	0.7 (0.9)	**Reference**	**Reference**
**HoNOS**	Absent (>2)	27% (3,256)	33,184	1.7 (1.7)	1.1 (1)	0.78 (0.01)^***^, [0.75, 0.8]	0.39 (0.01)^***^, [0.38, 0.4]
**ADL**	Present	73% (8,978)	83,535	1.5 (1.7)	0.4 (0.7)	**Reference**	**Reference**
**HoNOS**	Absent (>2)	33% (4,035)	36,291	1.6 (1.7)	0.9 (1)	0.91 (0.01)^***^, [0.89, 0.94]	0.75 (0.01)^***^, [0.73, 0.78]
**social**	Present	67% (8,199)	80,428	1.5 (1.7)	0.5 (0.8)	**Reference**	**Reference**
**HoNOS**	Absent (>2)	56% (6,891)	68,809	1.3 (1.6)	0.5 (0.8)	0.51 (0.01)^***^, [0.49, 0.52]	0.66 (0.01)^***^, [0.65, 0.68]
**positive**	Present	44% (5,343)	47,910	1.9 (1.8)	0.8 (0.9)	**Reference**	**Reference**
**HoNOS**	Absent (>2)	77% (9,387)	95,548	1.6 (1.7)	0.6 (0.8)	1.76 (0.02)^***^, [1.71, 1.82]	0.98 (0.02), [0.95, 1.01]
**depression**	Present	23% (2,847)	21,171	1.3 (1.6)	0.8 (1)	**Reference**	**Reference**
**Antipsychotic**	Yes	12% (1,500)	7,245	1.4 (1.6)	0.6 (0.8)	1.44 (0.01)^***^, [1.4, 1.47]	1.14 (0.01)^***^, [1.11, 1.17]
	No	88% (10,734)	109,474	1.6 (1.7)	0.6 (0.9)	**Reference**	**Reference**
**Total**		**100% (12,234)**	**116,719**	**1.6 (1.7)**	**0.6 (0.9)**		

* p < 0.05, **p < 0.01,

*** *p < 0.001*.

Several factors initially extracted (first language, antidementia medication, and antidepressant medication) included more than 30% of missing data and consequently were dropped from the analyses.

Furthermore, several categories were grouped to maximize association and/or reduce imbalance. For education, patients having a General Certificate of Secondary Education (GCSE), A-levels or University degrees were grouped together versus patients with no recorded education; for ethnicity, patients of non-white background were grouped in one category.

Results using NLP-CI indicate that patients with at least one symptom recorded are more likely to be male, of non-white background, living alone, and unemployed, which is broadly consistent with previous research in British aging cohorts (38). Additionally, the presence of cognitive symptoms was associated with problems with daily living, social impairment, and positive symptoms, with a potentially protective impact for antipsychotic medication. Patients with comorbid dementia were also more likely to show cognitive dysfunctions, supporting results from Bendayan et al. (36). The association of age with cognition appears inconclusive, and needs to be further disentangled by individual trajectories analysis.

HoNOS-CI shows a similar profile with differences for age and education, possibly due to coverage discrepancies (HoNOS is not used for children, and includes slightly different cognitive domains compared to the NLP application). Specifically, looking at a detailed breakdown of cognitive symptoms by education level (Table 4), attention and social cognition stand out as being more prevalent for patients with a qualification of GCSE or above compared to those without. This is further supported by the higher number of symptoms recorded for patients under the age of 40 in the unstructured portion of EHRs, and could be explained by the fact that certain cognitive dysfunctions, such as attention disorders, are more likely to be detected and dealt with in educational settings (39).

**Table 4:** Prevalence of different NLP-CI symptoms grouped by education.

**Education**	**Number in sample**	**NLP-CI avg**	**HoNOS-CI avg**	**Attention**	**Emotion**	**Executive function**	**Memory**	**Other**
None recorded	43% (5,290)	1.3 (1.6)	0.7 (0.9)	56%	51%	70%	62%	54%
GCSE	8% (944)	1.6 (1.7)	0.6 (0.9)	66%	62%	74%	69%	61%
A-level	24% (2,922)	1.7 (1.8)	0.5 (0.8)	66%	63%	71%	67%	60%
University	25% (3,078)	1.7 (1.8)	0.6 (0.8)	65%	64%	69%	66%	61%

### Association With Clinical Outcome (Number of Hospital Admissions and Length of Stay)

**Figure 2:**
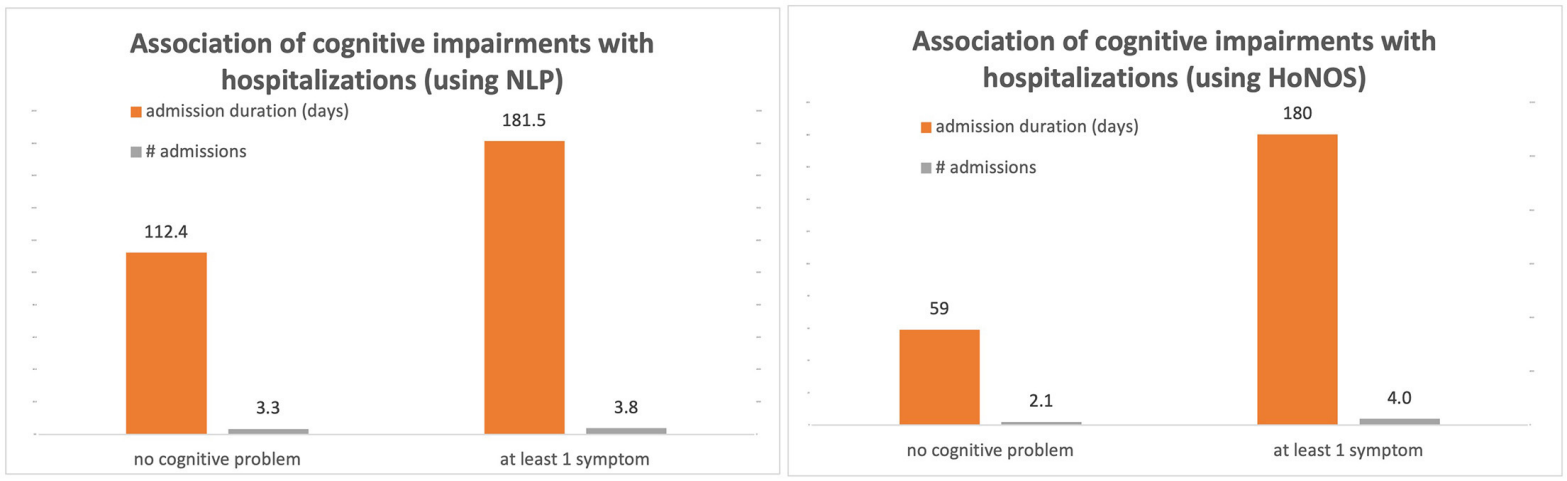
Association of HoNOS and NLP scores with mental health stays.

Table 5 and Figure 2 highlight the association of cognitive impairments with the number of hospital admissions and length of stay, within 6 months of mention of the problem. Regression analyses suggest that cognitive symptoms are associated with a higher probability of admission, as well as a longer length of hospitalization.

**Table 5:** Association between number of cognitive impairments (estimated using NLP and HoNOS) with mental health hospital admission and duration of admission between 2007 and 2020 (Sample B).

	**Number of ward admissions^a^**	**Duration of inpatient admission (days)^a^**
	**β-coeff (SE), CI 95%**	**β-coeff (SE), CI 95%**
**Associations with 1 or more cognitive impairments (NLP-CI-b: binary variable)**		
(0) Unadjusted	0.49 (0)^***^, [0.49, 0.5]	23.41 (0.16)^***^, [23.1, 23.71]
(1) Model 0 + age and gender	0.4 (0.01)^***^, [0.39, 0.41]	18.6 (0.22)^***^, [18.16, 19.03]
(2) Model 1 + socio-demographics	0.3 (0.01)^***^, [0.29, 0.31]	15.97 (0.23)^***^, [15.52, 16.42]
(3) Model 2 + HoNOS ADL, social, positive, depression	0.26 (0.01)^***^, [0.25, 0.28]	14.43 (0.23)^***^, [13.97, 14.89]
**Associations with incremental number of cognitive impairments (NLP-CI: 5-points scale ordinal variable)^b^**		
(0) Unadjusted	0.22 (0)^***^, [0.22, 0.22]	10.18 (0.05)^***^, [10.09, 10.28]
(1) Model 0 + age and gender	0.21 (0)^***^, [0.21, 0.22]	9.9 (0.06)^***^, [9.79, 10.02]
(2) Model 1 + socio-demographics	0.2 (0)^***^, [0.19, 0.2]	9.56 (0.06)^***^, [9.44, 9.68]
(3) Model 2 + ADL, social, positive, depression	0.19 (0)^***^, [0.19, 0.19]	9.3 (0.06)^***^, [9.18, 9.42]
**Associations with 1 or more cognitive impairments (HoNOS-CI-b binary variable)**		
(0) Unadjusted	0.33 (0)^***^, [0.32, 0.34]	18.76 (0.19)^***^, [18.38, 19.14]
(1) Model 0 + age and gender	0.03 (0.01)^***^, [0.02, 0.04]	6.07 (0.25)^***^, [5.59, 6.55]
(2) Model 1 + socio-demographics	0 (0.01), [−0.01, 0.01]	5.14 (0.24)^***^, [4.67, 5.62]
(3) Model 2 + HoNOS ADL, social, positive, depression	0.04 (0.01)^***^, [0.02, 0.05]	5.21 (0.25)^***^, [4.73, 5.69]

*p < 0.05, **p < 0.01,

*** *p < 0.001*.

a *Linear regression based on admissions within 6 months of symptom measure or mention*.

b *β-coefficients are per one unit increase on the ordinal scale*.

Patients with at least one mention of cognitive problems in their medical record have on average 49% more hospital admissions within 6 months of the mention than those without, and the admission is on average 23 days longer.

These associations are mitigated by age, socio-demographic and clinical factors. Specifically, being female, employed, married or cohabitating, and not showing problems with daily living, relationships and positive symptoms reduces the frequency and length of hospitalizations. After full adjustment, the frequency of admissions is indeed reduced by more than half (patients with one or more cognitive problems have a 23% greater likelihood of admission) and the average length of stay by 9 days. Overall, results are broadly consistent between NLP-CI and HoNOS-CI.

### Cognitive Symptoms Trajectories

Finally, mixed linear models were used to compare the cognitive trajectories of patients with Sz (using sample A for NLP-CI and sample B for HoNOS-CI, in order to maximize the number of datapoints and capture the complete evolution as recorded in EHRs), and their associations with various socio-demographic and clinical factors.

Here, we use antipsychotic treatment as a proxy for positive symptoms, given HoNOS scores are not recorded as frequently as cognitive symptoms are mentioned in unstructured text and consequently do not provide meaningful association when using sample A. In order to compare HoNOS-CI with NLP-CI, the baseline is defined as the date when the patient has their first mention recorded (on average 42 years old for HoNOS and 39 for NLP-CI).

**Figure 3:**
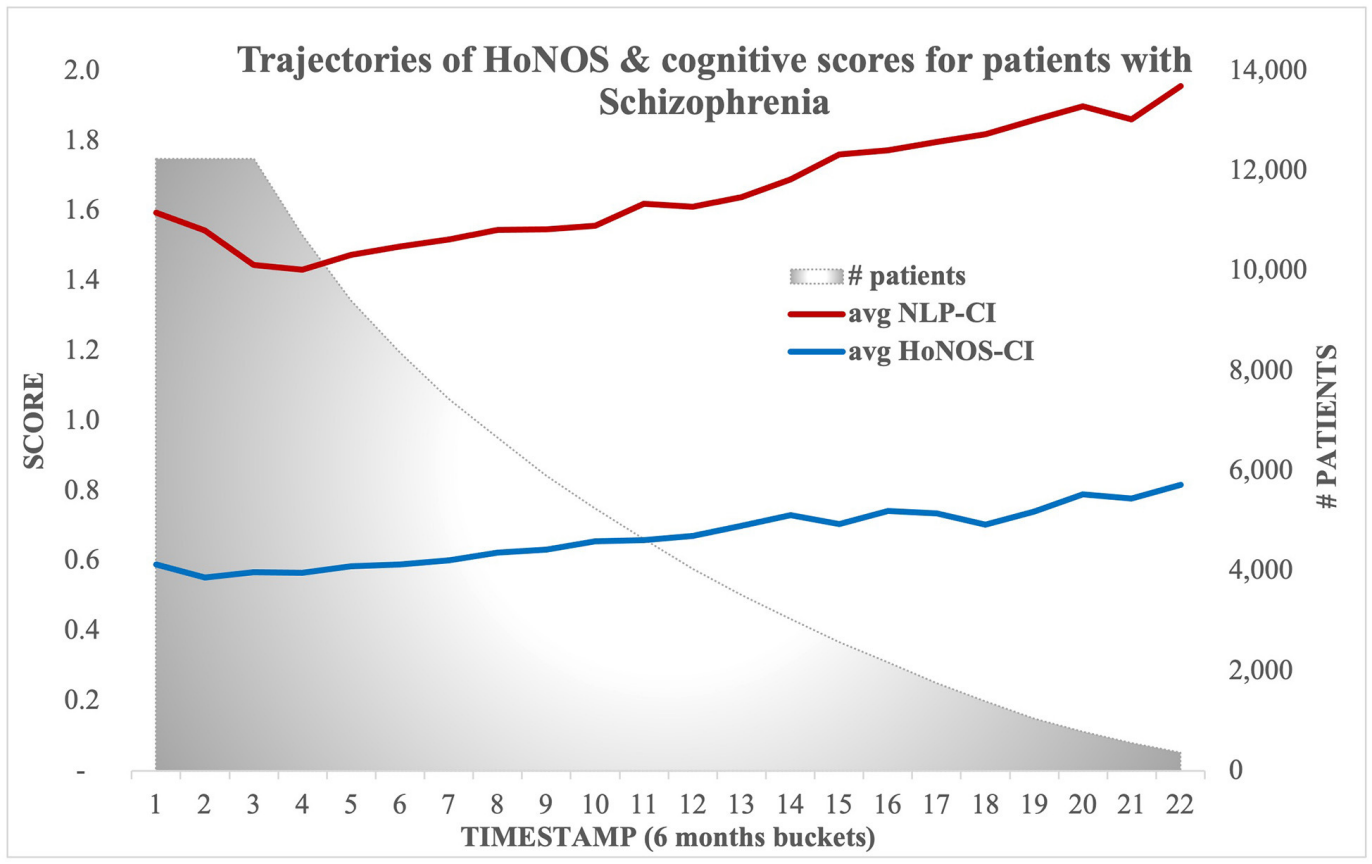
Trajectories of HoNOS and NLP-based Cognitive Impairment scores (Sample B).

Figure 3 illustrates the evolution of both scores over time, showing on average a constant slightly increasing slope.

Results obtained with mixed linear models, summarized in Tables 6, 7, confirm most associations found using regression models for NLP-CI, specifically that patients who are male, of non-white background, educated, and living alone are more likely to show higher cognitive impairments when admitted and/or a faster worsening of the problems over time. After full adjustment, patients have on average 1.32 cognitive symptoms mentioned in EHRs at baseline, and this number increases by 0.05 every 6 months, indicating that cognitive problems worsen with age (resp. a severity of 0.96 at baseline and an increase of 0.06 when using HoNOS-CI).

**Table 6:** Estimates for the fully adjusted linear mixed model for HoNOS-CI and NLP-CI trajectories.

	**NLP-CI (Sample A)**	**NLP-CI adj (Sample B)**	**HoNOS-CI (Sample B)**
	**Estimate (SE), CI 95%**	**Estimate (SE), CI 95%**	**Estimate (SE), CI 95%**
**Intercept**	1.32 (0.04)^***^, [1.24, 1.41]	0.93 (0.03)^***^, [0.87, 0.99]	0.96 (0.03)^***^, [0.91, 1.01]
Gender (female)	−0.04 (0.02), [−0.08, 0]	−0.02 (0.02), [−0.05, 0.01]	−0.05 (0.01)^***^, [−0.08, −0.03]
Has dementia (no)	0.01 (0.04), [−0.07, 0.09]	−0.05 (0.03), [−0.1, 0.01]	−0.34 (0.02)^***^, [−0.39, −0.3]
Education (GCSE or above)	0.6 (0.02)^***^, [0.56, 0.64]	0.33 (0.02)^***^, [0.3, 0.36]	−0.13 (0.01)^***^, [−0.16, −0.11]
Ethnicity (white)	−0.05 (0.02)*, [−0.09, −0.01]	−0.02 (0.02), [−0.05, 0.01]	0.01 (0.01), [−0.02, 0.03]
Married / cohabiting (yes)	−0.1 (0.04)**, [−0.18, −0.03]	−0.05 (0.03), [−0.11, 0]	−0.05 (0.02)*, [−0.1, −0.01]
Employed (yes)	0.11 (0.06), [−0.01, 0.22]	0.05 (0.04), [−0.03, 0.13]	−0.14 (0.03)^***^, [−0.2, −0.08]
Antipsychotic (yes)	−0.61 (0.03)^***^, [−0.66, −0.55]	−0.35 (0.02)^***^, [−0.39, −0.32]	−0.04 (0.01)**, [−0.06, −0.01]
**Slope linear**	0.05 (0.01)^***^, [0.05, 0.06]	0.04 (0.00)^***^, [0.03, 0.05]	0.06 (0.00)^***^, [0.05, 0.07]
Gender (female)	0.01 (0.00)*, [0, 0.01]	0.01 (0.00)**, [0, 0.01]	0.01 (0.00)**, [0, 0.01]
Has dementia (no)	−0.05 (0.00)^***^, [−0.06, −0.04]	−0.04 (0.00)^***^, [−0.04, −0.03]	−0.05 (0.00)^***^, [−0.06, −0.04]
Education (GCSE or above)	−0.02 (0.00)^***^, [−0.03, −0.02]	−0.02 (0.00)^***^, [−0.02, −0.01]	−0.01 (0.00)^***^, [−0.01, −0.01]
Ethnicity (white)	0.01 (0.00), [0, 0.01]	0 (0.00), [0, 0.01]	0 (0.00), [0, 0]
Married / cohabiting (yes)	−0.01 (0.00)**, [−0.02, −0.01]	−0.01 (0.00)**, [−0.01, 0]	0 (0.00), [−0.01, 0.01]
Employed (yes)	−0.01 (0.01)*, [−0.03, 0]	−0.01 (0.01), [−0.02, 0]	0 (0.01), [−0.01, 0.01]
Antipsychotic (yes)	0.05 (0.00)^***^, [0.04, 0.05]	0.03 (0.00)^***^, [0.02, 0.03]	0.01 (0.00)^***^, [0.01, 0.01]
**Variances**			
Intercept	0.95 (0.01)^***^	0.37 (0.01)^***^	0.32 (0.01)^***^
Slope	−0.07 (0.00)^***^	−0.03 (0.00)^***^	−0.02 (0.00)^***^
Residual	0.01 (0.00)^***^	0 (0.00)^***^	0 (0.00)^***^
**Fit statistics for linear models**			
BIC			
−2LL	−384,229	−257,483	−130,724

*p < 0.05, **p < 0.01,

*** *p < 0.001*.

**Table 7:** Slope estimates for the fully adjusted linear mixed model for HoNOS-CI and NLP-CI trajectories.

	**NLP-CI (Sample A)**	**NLP-CI adj (Sample B)**	**HoNOS-CI (Sample B)**
	**Estimate (SE), CI 95%**	**Estimate (SE), CI 95%**	**Estimate (SE), CI 95%**
0. Unadjusted	0 (0.00), [0, 0]	0.01 (0.00)^***^, [0.01, 0.01]	0 (0.00), [0, 0]
1. Model 0 + age and gender	0 (0.00), [0, 0]	0.01 (0.00)^***^, [0, 0.01]	0.01 (0.00)^***^, [0.01, 0.02]
2. Model 1 + socio-demographics	0.05 (0.01)^***^, [0.05, 0.06]	0.05 (0.00)^***^, [0.04, 0.05]	0.06 (0.00)^***^, [0.06, 0.07]
3. Model 2 + antipsychotics	0.05 (0.01)^***^, [0.04, 0.06]	0.04 (0.00)^***^, [0.03, 0.05]	0.06 (0.00)^***^, [0.05, 0.07]

*** *p < 0.001*.

The comparison between HoNOS-CI and NLP-CI models further informs the association of education and age with cognitive impairments. Both NLP and HoNOS scores indicate a slower decline in cognitive function for patients with education. However, educated patients have on average 5% more mentions of cognitive problems in the free text at baseline (which likely corresponds to when they are students). Again this may be explained by the greater coverage of cognitive domains and age groups with the NLP application, which could therefore potentially allow earlier detection of impairments, specifically for attention and emotion. Sensitivity analyses showed that removing “attention” and “emotion” from the cognitive domains covered (defining the new metric “NLP-CI adj”) and including patients only present in sample B attenuated the impact of education on the trajectories.

## Discussion

### Strengths and Limitations

Using an NLP method to extract symptoms from EHRs proves to be an effective and robust method, which does not require any specific training or wide-scale patient recruitment to take part in lengthy and costly tests. Our results show that cognitive impairments are frequently mentioned in medical records, and suggest associations generally in line with the HoNOS cognitive subscale. However, cognitive problems prove difficult to detect and assess, and are less routinely mentioned than positive symptoms, for which treatment remains a high priority in the British mental health system, and to a lesser extent negative symptoms (40). Furthermore, we assume that an absence of documentation represents an absence of symptoms, but another possibility could be that cognitive dysfunctions are secondary to positive symptoms and less frequently recorded. This has been partially mitigated in our study by the inclusion of positive symptoms as a covariate, but nonetheless may still result in underestimating the prevalence of cognitive problems when using the information extracted from free text compared to direct assessments.

Another limitation of our analysis was the use of a fixed set of keywords to identify symptoms, which could potentially exclude certain patients from detection, however, using a “free” approach would necessitate the annotation of a very large number of documents. Moreover, the keywords were derived from two major terminology sources (26, 27), which should cover most terms used by clinicians in medical records. This is further mitigated by the fact that the list was reviewed by clinicians and enriched by most common misspellings detected in CRIS electronic records [following the method proposed in Viani et al. (41)].

A key strength of our approach is the large sample covered, representative of patients with schizophrenia as well as the overall CRIS population. Using NLP allows us to rapidly extract information on large datasets, and the keywords approach proves generalizable for various types of symptoms and disorders (18, 23). This contrasts with most clinical assessments or rating scales, which are not often administered, or may not always capture all types of impairments. For instance HoNOS is routinely administered in the UK but is limited in its coverage of cognitive domains and population (34, 35). Despite these differences, the data extracted using the NLP framework shows a strong correlation with the HoNOS cognitive subscale in terms of symptoms trajectories, association with socio-demographic and clinical variables. We found cognitive dysfunction to be a common feature of patients with Sz, and to have a clear association with adverse clinical outcomes. Our data also highlights that cognitive impairments are an important driver for hospital admission, which is more traditionally linked to positive (42) or negative (18) symptoms.

Finally, the discrepancies related to age and education could indicate that clinicians perceive and document cognitive dysfunctions more readily when someone has previously completed a university degree but subsequently developed psychosis (assuming this occurred after university). This should be further explored in order to better assess the relationship between education and early detection of cognitive problems, in particular for attention and emotional impairments.

In summary, whilst NLP methods cannot provide results as accurate as clinical assessments or rating scales, they present the advantage of being cost-effective and cover a larger population as well as a broader and more granular timespan. Consequently, NLP results can be seen as complementary and used to screen patients showing cognitive problems, who could in turn benefit from more specific tests (e.g., by being selected to take part in neurocognitive batteries studies) as well as targeted treatment.

### Future Research and Conclusions

Our findings suggest that cognitive impairments can reliably be extracted from clinical records using NLP methods, and reveal their high prevalence in patients with Sz as well as a clear association with poor clinical outcomes.

Analyzing data from the free text of EHRs provides broad coverage in terms of the number of patients and age range, in contrast to neurocognitive batteries or rating scales. Therefore, this can facilitate systematic patient screening and large scale and early detection of cognitive problems. Furthermore, NLP tools allow us to extract a number of socio-demographic and clinical parameters not recorded in the structured portion of EHRs which, combined together, can provide new insights into the onset and development of symptoms and eventually inform clinical decisions.

This reveals the potential of such automated tools to harvest meaningful information from the unstructured text of medical records, and in this specific study highlights the importance of developing more targeted and effective treatments for patients with Sz suffering from cognitive impairments.

## Data Availability Statement

The datasets presented in this article are not readily available because they contain participants identifiable data. Requests to access the datasets should be directed to aurelie.mascio@kcl.ac.uk.

## Author Contributions

AM conceived the study, conducted the data analysis, and drafted the manuscript. RS, AR, RP, and TP participated in the study design and interpretation of the results. RB, MW, and LM helped design the annotation guidelines and annotated the clinical data. All authors commented on drafts of the manuscript and approved the final version.

## Conflict of Interest

The authors declare that the research was conducted in the absence of any commercial or financial relationships that could be construed as a potential conflict of interest.
